# Improvement in Cervical Spinal Alignment and Posture May Redefine Recovery Pathways for Motor Vehicle Collision Whiplash Injury: A Multicenter Retrospective Consecutive Case Series

**DOI:** 10.3390/healthcare14030373

**Published:** 2026-02-02

**Authors:** Michael L. Underhill, Curtis A. Fedorchuk, Cole G. Fedorchuk, Douglas F. Lightstone

**Affiliations:** 1 Private Practice, Beaverton, OR 97006, USA; 2 Institute for Spinal Health and Performance, Cumming, GA 30041, USA

**Keywords:** chiropractic biophysics, traffic collision, whiplash injury, cervical spine, lordosis, posture, neck pain, rehabilitation

## Abstract

**Background/Objectives:** Motor vehicle collision (MVC) cervical acceleration–deceleration (CAD) spine injuries are prevalent, costly, and complicated conditions. CAD injuries, or whiplash-associated disorders (WAD), present with neuromusculoskeletal signs and symptoms. In total, 50% of MVC WAD/CAD injuries result in chronic neck-related disability, of which 30% are moderate-to-severe. Poor recovery is associated with little-to-no recovery after 3 months. This study reports on health outcomes of patients with MVC/CAD injuries treated with Chiropractic BioPhysics^®^ (CBP^®^) spinal rehabilitation beyond little-to-no recovery in neck pain (NP) and disability after 3-to-4 months. **Methods:** This multicenter retrospective consecutive series reports on patients who met inclusion/exclusion criteria from a records review from two private practices with advanced training in CBP^®^. **Results:** In total, 51 patients (26 males), 18–74 years-of-age (mean age 42.8 ± 3.6 years), presented with post-MVC NP and disability. Pre-treatment radiographs revealed decreased cervical curvature (ARA C2-C7) measuring −10.3 ± 2.0° (ideal is −42.0°) and anterior head translation (Tz C2-C7) measuring 28.5 ± 2.0 mm (ideal is 0 mm). The pre-treatment NP numeric rating scale (NRS) scored 6.0 ± 1.0, and the neck disability index (NDI) scored 54.3 ± 9.3% (severe). Patients were treated using CBP^®^ for 64.5 ± 4.7 visits over 31.6 ± 3.7 weeks. Post-treatment radiographs revealed an improved ARA C2-C7 to −22.5 ± 2.3° and Tz C2-C7 to 15.9 ± 1.6 mm (*p* < 0.001). Subsequent 3-to-4-month re-exam showed little-to-no change in NP and disability outcomes. Post-treatment outcomes at a mean 18.5 weeks after the 3-to-4-month re-exam showed significant (*p* < 0.001) improvements in NP NRS to 1.1 ± 0.7 and NDI to 6.8 ± 5.5 (minimal). **Conclusions:** CBP^®^ improves cervical lordosis and posture, which may help improve moderate-to-severe WAD/CAD spine injuries beyond 3-to-4 months of little-to-no recovery.

## 1. Introduction

Neck pain (NP) is a prevalent condition that leads to substantial pain, disability, and economic strain. It affects individuals, their families, the healthcare system, and national economies [1]. In 2016, among 154 health conditions, NP represented the highest share of healthcare expenditures in the U.S., totaling approximately $134.5 billion. By 2019, there were 222.7 million existing cases of NP worldwide, along with 47.5 million new cases, and 22.1 million years lived with disability (YLD) documented, with these figures continuing to rise [2].

Cervical acceleration–deceleration (CAD) spine injuries are prevalent, costly, and complicated conditions, usually caused by trauma sustained in a motor vehicle collision (MVC). CAD injuries present with musculoskeletal signs and symptoms, including pain, stiffness, tenderness of the neck and/or shoulder(s), and decreased range of motion (ROM), and neurological symptoms, including headache, dizziness, referred or radicular pain, numbness, and/or weakness from the cervical spine [3,4]. Collectively, CAD injuries are known as whiplash-associated disorders (WAD). In people who sustain WAD/CAD injuries following a motor vehicle collision (MVC), approximately 50% of individuals will experience ongoing, long-term, chronic neck-related disability [5,6], and 30% will experience ongoing, long-term, chronic neck-related moderate-to-severe disability [7,8]. Poor recovery has been consistently reported to be associated with moderate-to-high initial neck pain intensity and neck-related disability and little-to-no change in recovery status after 3 months [5,6].

In 2006, Harrison et al. analyzed and reported on sagittal cervical radiographs of 41 consecutive patients who experienced MVC. Post-MVC neutral lateral cervical (NLC) spine radiographs were compared with NLC radiographs taken within 30 days prior to the MVC. The study revealed a significant loss of cervical curvature along with a change in the sagittal alignment geometry referred to as a second-order buckling configuration in the context of snap-through buckling of columns [9]. Additionally, studies have highlighted the importance of sagittal cervical spinal alignment and posture [9,10] and the importance of correcting cervical spinal alignment and posture following MVC WAD/CAD injuries [9,11]. This raises the question of whether improving cervical spinal alignment and posture can have a positive impact on patients with WAD/CAD injuries who experience little-to-no change in recovery after 3 months.

The objective of this case series is to report on improved functional, symptomatic, and radiographic measures of the cervical spine in patients with moderate-to-severe NP and disability from MVC WAD/CAD injuries following structural spinal rehabilitation of the cervical spine in patients who experienced little-to-no change in recovery after 3 months of prior rehabilitation.

## 2. Methods

### 2.1. Patients

This multicenter retrospective consecutive case series involved a records review of patients and documenting the clinical results of all patients who met the inclusion/exclusion criteria. There is no control group. This study was confirmed for institutional review board (IRB) exemption by Advarra IRB, registered with OHRP and FDA under IRB#00000971 on 26 February 2025, according to Exemption Category 4: secondary research of identifiable private information for which consent is not required due to information recorded in such a manner that the identity of the human subjects cannot readily be ascertained directly or through identifiers linker to subjected, the investigator will not contact the subjects, and the investigator will not re-identify subjects. Informed consent was waived because of the retrospective nature of the study.

Patients were selected from a records review from two private practices with advanced training in the structural rehabilitation of the spine and that follow Chiropractic BioPhysics^®^ (CBP^®^) protocols [12]. The patients selected for this case series met the following inclusion criteria:

1. Patients were adults with a minimum age of 18 years;
2. Health history revealed the patient was involved in a MVC within 1 month of starting CBP^®^ care and experienced WAD/CAD injury-associated NP and disability;
3. Physical examination revealed NP (International Classification of Diseases, Tenth Revision (ICD-10) M54.2), abnormal cervical posture (ICD-10 R29.3), reduced neck mobility (ICD-10 Z74.09), cervical spine dysfunction (ICD-10 M99.01), and/or traumatic spondylopathy of the cervical region (ICD-10 M48.32);
4. Pre-treatment patient-reported outcomes (PRO) using the NP numeric rating scale (NRS) and neck disability index (NDI) revealed moderate-to-severe NP and disability;
5. NLC radiographs including C2 to C7 revealed loss of cervical lordosis (ICD-10 M95.3);
6. At least 3 months of rehabilitation where patients experienced little- to- no change in recovery status following the MVC, using minimal clinically important differences (MCID) for PROs as thresholds for change;
7. Compliance with treatment recommendations, including CBP^®^ spinal rehabilitation, Mirror Image^®^ (MI) chiropractic adjustments, therapeutic spinal exercises, and mechanical spinal traction, followed by post-treatment NLC radiographs for comparison to pre-treatment radiographs.

Patients were not selected for this case series if they met certain exclusion criteria:

1. Presence of red flags or contraindications for chiropractic adjustments, therapeutic spinal exercises, or mechanical spinal traction to the cervical spine;
2. Presence of cervical or cervicothoracic scoliosis or lateral translations of C2 with respect to T4 (Tx C2-C4) measuring 7 mm or greater [13].

All methods were carried out according to the relevant guidelines and regulations. This case series is a retrospective analysis of patients treated in a private practice according to standards of care. This case series did not involve any experimentation on human or animal subjects, and all data and identifying content have been anonymized. Such a case series involves activities that do not meet the Department of Health and Human Services definition of research requiring institutional review board (IRB) approval. As such, this study does not have an IRB.

### 2.2. Patient-Reported Outcomes

PROs offer essential insights into the symptoms and health conditions experienced by patients. These outcomes can be expressed in qualitative terms (e.g., mild, moderate, severe, etc.) or quantitative metrics (e.g., scores or percentages ranging from 0 to 10 or 0 to 100). Patient-reported outcome measures (PROMs) are the specific tools used to assess these PROs, which encompass aspects such as functional status, symptoms, and the overall burden of those symptoms related to various health issues [14].

#### 2.2.1. Neck Pain Numeric Rating Scale

The NP NRS is a PROM that enables individuals to assess the intensity of their NP on a scale from 0 to 10, where 0 indicates no pain, and 10 signifies the highest level of severity. The NP NRS is used during pre-treatment, a progress re-exam at 3-to-4 months, and post-treatment evaluations. To demonstrate a clinically meaningful improvement in pain levels, the minimally clinically important difference (MCID) is established at −2.2 points on the scale [15].

#### 2.2.2. Neck Disability Index

The NDI evaluates how NP affects activities of daily living (ADLs) and quantifies the degree of disability on a percentage scale from 0 to 100, with 0 indicating no disability from neck pain and 100 representing total disability. The NDI is utilized during pre-treatment, a progress re-exam at 3-to-4 months, and post-treatment evaluations. A change of 15% in the NDI score is regarded as clinically significant [16].

### 2.3. Radiographic Analysis

Spinal radiographs are essential for identifying potential red flags or contraindications to spinal treatments, assessing pathologies, spinal alignment, and vertebral subluxations. This information helps determine the appropriate strategies for structural spinal rehabilitation and monitors patient progress within a spinal correction program [17].

The Harrison posterior tangent method is used to measure cervical angles obtained by drawing a line that is tangent to the posterior edges of each vertebral body from C2 to C7. NLC radiographs were evaluated using PostureRay^®^ Electronic Health Records (EHR) Software version 26 (PostureCo, Inc., Trinity, FL, USA), following the Harrison posterior tangent method for sagittal plane spine assessments. Radiographs were measured by two doctors with advanced training in CBP methods and CBP instructor status, with 40 and 10 years of experience. The examiners were not blinded to the assessments they were performing, but measurements were completed several months apart from pre-treatment radiographs, which were not referenced prior to measuring the post-treatment radiographs. Intra- and inter-examiner reliability was not assessed. Cervical lordosis is measured using the posterior tangents of C2 to C7, providing the absolute rotational angle (ARA C2-C7) and sagittal translation (in the *z*-axis) of C2 with respect to C7 (Tz C2-C7). These examination and analysis methods are valid [18,19,20,21,22], reliable, and repeatable [18,19,23,24,25,26], as is posture [18].

Pre-CBP^®^ treatment radiographs were taken at initial examination prior to any treatment intervention(s). Post-CBP^®^ treatment radiographs were taken at least 24 h after completion of treatment. This approach is employed to rule out short-term effects of spinal rehabilitation on spinal alignment and posture. Patient positioning was consistent with procedures taught in CBP^®^ technique, which are very reliable and repeatable [17]:

For lateral cervical radiographs, the patient’s shoulders were positioned perpendicular to the radiographic bucky, and the patient was instructed to close his/her eyes, to flex and extend the head twice, and come to a resting neutral position. This neutral resting posture is that in which the patient perceives his/her head to be looking straight forward. The patient then opens his/her eyes and is instructed to look straight ahead without moving. The patient’s abnormal sagittal plane posture is left as is (i.e., it is not guided toward an ideal neutral position). The lateral cervical is taken at the standard tube distance of 182.9 cm (72 inches), with the central ray located approximately at the C4 level [17].

For spinal postural and radiographic analysis, a right-hand, thumb-up Cartesian coordinate system is employed to represent translations and rotations around the x, y, and z-axes of the head, thorax, and pelvis. Positive or negative values indicate the direction of translation in or rotations around these axes. The notation indicates translation (T) or rotation (R), the axis (x, y, or z), and alphanumeric designations for head (H), thorax (T), pelvis (P), or specific vertebrae [12].

### 2.4. Interventions and Outcomes

The treatment visits for the patients consisted of MI corrective adjustments, therapeutic spinal exercises, and mechanical spinal traction per CBP^®^ protocols [12] administered at two CBP spinal rehabilitation clinics by two doctors with advanced training in CBP methods and CBP instructor status with 40 and 10 years of experience. MI refers to the corrective or over-corrective positioning of the spine during spinal adjustments, therapeutic exercises, and mechanical traction to normalize spinal alignment and biomechanics [12]. CBP^®^ protocol approaches spinal rehabilitation based on the history, physical examination, and radiographic imaging findings of the patient. The CBP^®^ treatment visits for the patients in this series consisted of the following MI corrective adjustments, therapeutic spinal exercises, and mechanical spinal traction as determined from their initial findings.

**Figure 1:**
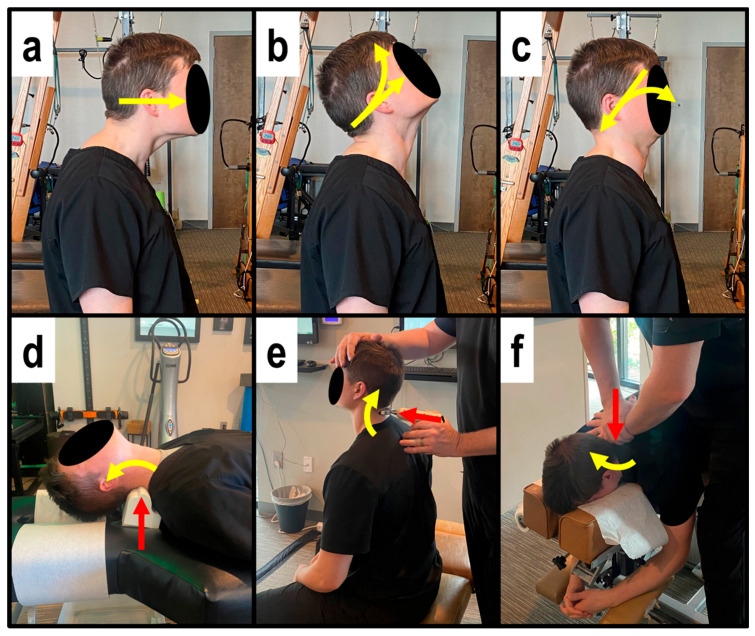
(**a**–**f**). The red arrow indicates the vector of corrective force (in this figure, the force is the MI corrective adjustment), and the yellow arrow indicates the Mirror Image (MI) corrective motion of the patient. (**a**–**c**) The three steps of the Fedorchuk cervical exercises to induce a cervical lordosis; (**d**) supine cervical extension traction with a posterior-to-anterior (P-A) fulcrum at the mid-neck; (**e**) seated corrective adjustment involving a P-A adjusting instrument-assisted thrust with the neck in extension; (**f**) prone corrective adjustment on an adjusting table involving a P-A manual thrust with the neck positioned into extension.

MI therapeutic spinal exercises strengthen weak musculature that has adapted to unhealthy posture to help maintain spinal corrections [12]. MI exercises involved the patient performing Fedorchuk cervical exercises to induce a cervical lordosis [27,28] involving:

1. Maximum anterior head translation (+TzH) (Figure 1a);
2. While maintaining +TzH, maximum head extension (−RxH) (Figure 1b);
3. While maintaining the −RxH, posterior head translation (−TzH) (Figure 1c) [27,28].

The patient held the final position for 10 s before relaxing and repeating for 3 sets of 20 repetitions.

MI mechanical spinal traction allows for viscoelastic plastic deformation of spinal ligaments [12] by initiating muscle and ligament creep for long-term spinal and postural correction. MI traction was performed using a Cervical Denneroll^TM^ Spinal Orthotic (Denneroll^TM^ Spinal Orthotics, Wheeler Heights, NSW, Australia) as a fulcrum behind the neck at the mid-to-lower cervical spine (C5-C6) to induce cervical extension. The cervical Denneroll lies flat on the ground or table, creating a vertical fulcrum angle at the point of contact. MI traction was performed for periods starting at 3 min at the initial treatment session and increasing by 2 min with each subsequent treatment session until 15 to 20 min was reached, which was maintained at each visit until the completion of treatment (Figure 1d).

MI corrective adjustments stimulate mechanoreceptors and proprioceptors to help the body adapt to normal, healthy posture [12]. MI adjustments were delivered at each visit while seated or prone using an OMNI elevation adjusting table with sectional drop-mechanisms and setting up the patient in the position obtained during MI position followed by a posterior-to-anterior (P-A) thrust to the mid-to-lower cervical vertebrae by hand (Figure 1e) and also using the Impulse^®^ Adjusting Instrument (Neuromechanical Innovations, Chandler, AZ, USA) (Figure 1f).

### 2.5. Statistical Analyses

The primary outcomes were ARA C2-C7, Tz C2-C7, NP NRS, and NDI. Before pooling the data, baseline characteristics were summarized at the patient level, including sex, height, weight, age, number of treatment visits, and duration of treatment.

A correlation analysis was performed on all outcome variables to assess whether changes in one variable would correspond to changes in another [29]. Understanding these relationships is important, as highly correlated variables would not be included in multivariable analyses. The correlation coefficient quantitatively measures the direction and strength of how variables may vary together, with Pearson’s correlation coefficient utilized for this analysis. The results are visualized in a heatmap where darker colors indicate stronger positive or negative correlations.

Descriptive statistics, including mean and standard deviation (STD), were calculated for all outcomes. Two-sample *t*-tests were conducted to identify statistically significant differences between pre- and post-CBP^®^ treatment results [30]. Additionally, one-sample *t*-tests were performed to compare NP NRS and NDI against the minimal clinically important difference (MCID), defined as the smallest quantitative change perceived by patients that would warrant a change in the clinical status of the patient. This patient-centered measure reflects both the magnitude of change and its perceived value. The strength of these comparisons was assessed using statistical significance, indicated by the *p*-value at a 95% confidence interval [31].

Moreover, post hoc power analyses were carried out based on the primary outcome results. These analyses help evaluate the adequacy of the sample size used in the study, although they do not alter the results or conclusions drawn [32].

All the summaries and statistical analyses were performed using the current version of the open-source statistical programming language SAS 9.4. Summary statistics used PROC FREQ/PROC MEANS, *t*-tests used PROC TTEST, descriptive statistics used PROC ANOVA, correlation analysis used PROC CORR, and the multivariable linear model for pre-post analysis used PROC GLM. The symbol ± indicates standard deviation (SD).

## 3. Results

**Figure 2:**
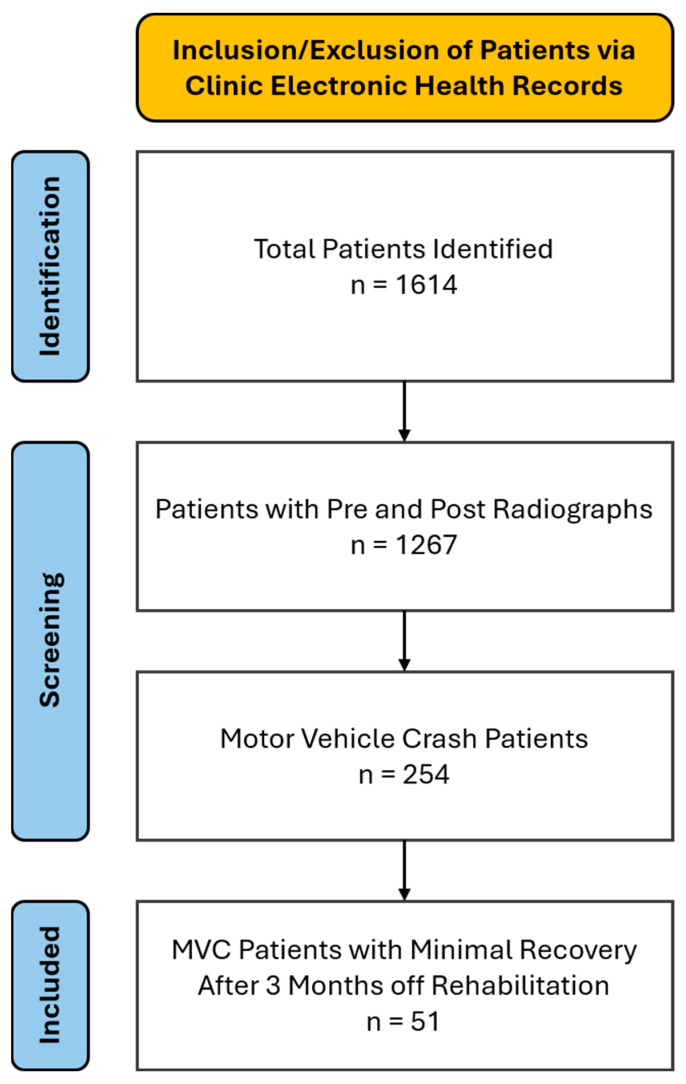
Basic flowchart illustrating the patients identified, screened, and included from the clinics, and the patients included based on the inclusion criteria.

The baseline characteristics of the 51 patients included in the study are summarized in Table 1. The patient population comprised 51 patients (25 females and 26 males) with a mean age of 42.8 ± 3.6 years (range of 18 to 74 years), height of 171.0 ± 2.6 cm, and weight of 84.7 ± 9.4 kg (Table 1) who presented with a chief complaint of NP with associated neck and upper trapezii stiffness and reduced ADL function. Figure 2 is a basic flowchart illustrating the patients identified, screened, and included from the clinics, and the patients included based on the inclusion criteria.

### 3.1. Pre-Treatment

**Figure 3:**
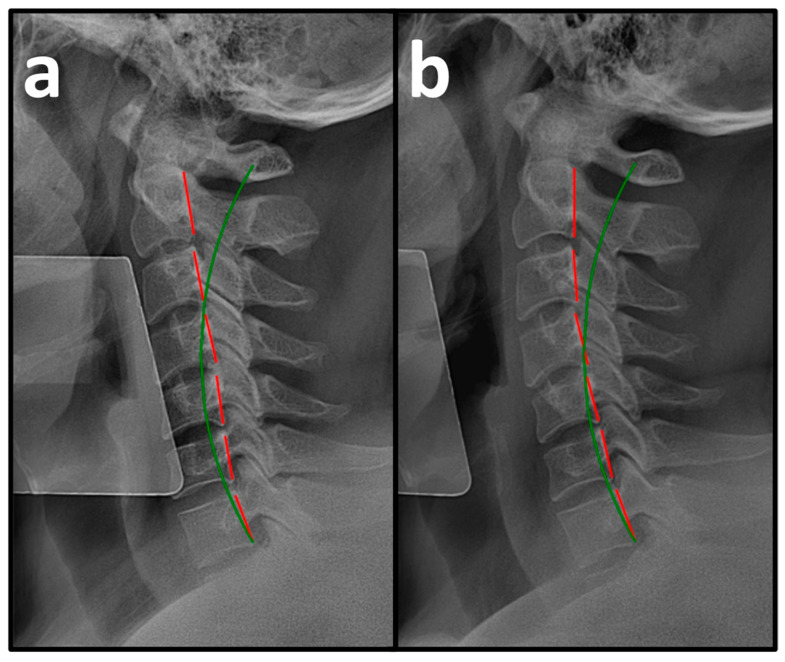
(**a**,**b**) Pre-treatment and post-treatment neutral lateral cervical (NLC) radiographs of a 30-year-old male initially reporting 5/10 neck pain (NP) and 50% (severe) disability due to NP. The green line represents a normal, ideal sagittal cervical alignment, and the red lines represent the actual posterior tangent lines of the C2 to C7 vertebrae. (**a**) Pre-CBP^®^ treatment NLC radiograph with an absolute rotational angle (ARA) C2-C7 measuring −7.4° (ideal is −42°) and sagittal translation (Tz) C2-C7 measuring 21.4 mm. (**b**) Post-CBP^®^ treatment NLC radiograph after 61 visits over 23 weeks with improvements in ARA C2-C7 to −24.8° and Tz C2-C7 to 18.7 mm.

**Figure 4:**
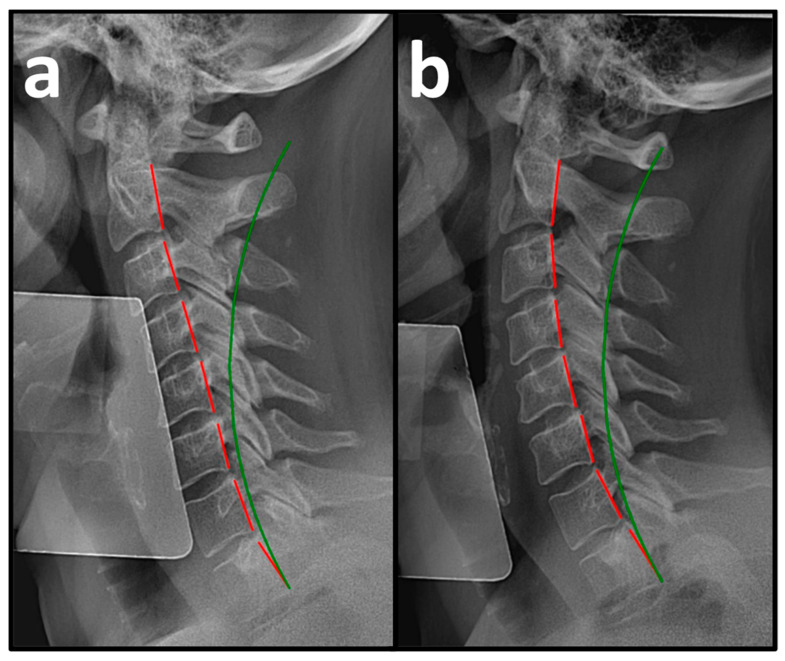
(**a,b**) Pre-treatment and post-treatment neutral lateral cervical (NLC) radiographs of a 47-year-old male initially reporting 7/10 neck pain (NP) and 46% (moderate) disability due to NP. The green line represents a normal, ideal sagittal cervical alignment, and the red lines represent the actual posterior tangent lines of the C2 to T1 vertebrae. (**a**) Pre-CBP^®^ treatment NLC radiograph with an ARA C2-C7 measuring −15.4° (ideal is −42°) and Tz C2-C7 measuring 46.9 mm. (**b**) Post-CBP^®^ treatment NLC radiograph after 90 visits over 63 weeks with improvements in ARA C2-C7 to −28.2° and Tz C2-C7 to 28.9 mm.

**Figure 5:**
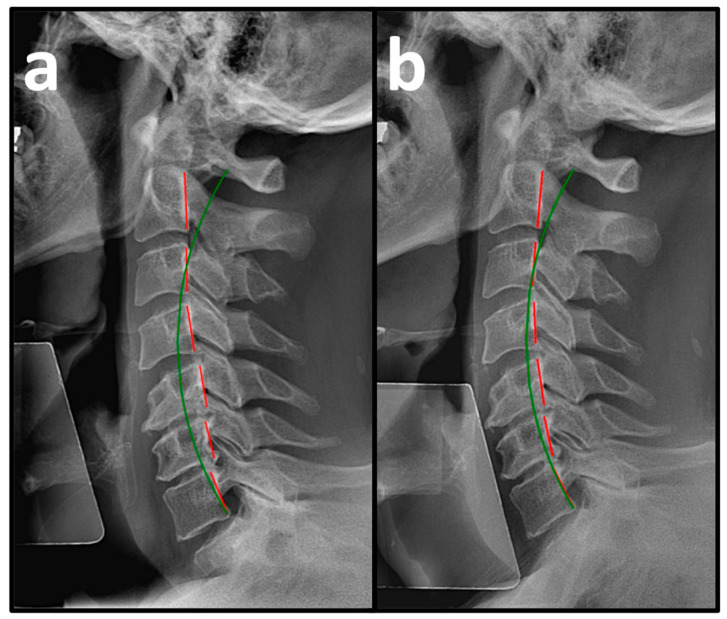
(**a**,**b**) Pre-treatment and post-treatment neutral lateral cervical (NLC) radiographs of a 60-year-old female initially reporting 6/10 neck pain (NP) and 52% (severe) disability due to NP. The green line represents a normal, ideal sagittal cervical alignment, and the red lines represent the actual posterior tangent lines of the C2 to C7 vertebrae. (**a**) Pre-CBP^®^ treatment NLC radiograph with an ARA C2-C7 measuring −14.0° (ideal is −42°) and Tz C2-C7 measuring 22.5 mm. (**b**) Post-CBP^®^ treatment NLC radiograph after 90 visits over 34 weeks with improvements in ARA C2-C7 to –24.8° and Tz C2-C7 to 17.6 mm.

Pre-treatment PROs revealed a mean NP NRS score of 6.0 ± 0.3, indicating moderate NP, and a mean NDI score of 54.3 ± 9.7%, indicating severe disability due to NP (Table 2). Pre-treatment NLC radiography revealed a mean Tz C2-C7 measuring 28.5 ± 7.1 mm (ideal is 0 mm and average is 15 mm) and mean ARA C2-C7 measuring −10.3 ± 7.0° (normal is −42° and average is 34°) (Figure 3a, Figure 4a and Figure 5a, Table 3) [30].

### 3.2. Post-Treatment

After a mean of 64.5 ± 4.7 treatment visits over 31.6 ± 3.7 weeks at a rate of 2.2 ± 0.2 treatment visits per week (Table 1), the patients reported improvements in NP NRS to 1.1 ± 0.7 (indicating minimal-to-mild NP) and NDI to 6.8 ± 5.5% (indicating minimal disability due to NP) (Table 2). Post-treatment NLC radiographs revealed improvements in mean Tz C2-C7 to 15.9 ± 2.3 mm and mean ARA C2-C7 to −22.5 ± 8.1° (Figure 3b, Figure 4b and Figure 5b, Table 3).

### 3.3. Pre-Treatment to Post-Treatment Radiographic Analysis

**Figure 6:**
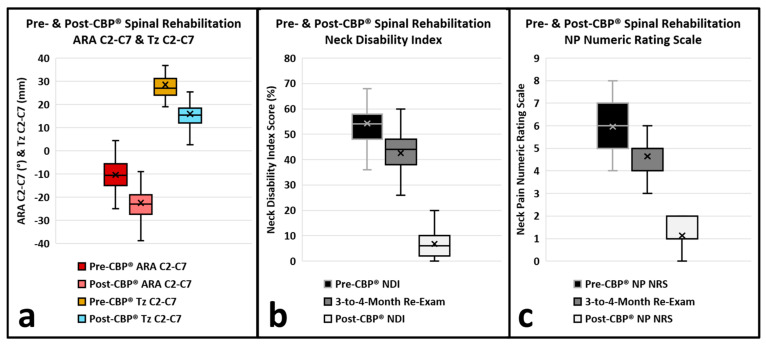
(**a**–**c**). Distribution of primary (ARA C2-C7 and Tz C2-C7) and secondary (NP NRS and NDI) outcomes of pre- and post-CBP^®^ structural spinal rehabilitation through box plots. (**a**) ARA C2-C7 and Tz C2-C7 improved from pre- to post-CBP^®^ structural spinal rehabilitation; (**b**) NDI did not reach MCID improvements from pre-treatment to 3-to-4-month re-exam. NDI did reach MCID improvement from 3-to-4-month re-exam to post-treatment and from pre-treatment to post-treatment; (**c**) NP NRS did not reach MCID improvements from pre-treatment to 3-to-4-month re-exam. NDI did reach MCID improvement from 3-to-4-month re-exam to post-treatment and from pre-treatment to post-treatment.

Changes in cervical curvature and translation following CBP^®^ treatment are outlined in Table 2. The ARA C2-C7 showed a significant increase post-treatment. The mean pre-treatment ARA (SD) was −10.3° (±7.0), while the post-treatment ARA increased to −22.5° (±8.1) with a mean difference of −12.2° (±4.3) (*p* < 0.001) (Figure 6a). The Tz C2-C7 showed a significant decrease post-treatment. The mean pre-treatment Tz (SD) was 28.5 mm (±7.1), while the post-treatment Tz decreased to 15.9 mm (±5.5) with a mean difference of −12.6 mm (±4.2) (*p* < 0.001) (Figure 6a).

In addition, the post hoc power analyses were conducted based on the output for primary outcomes. The powers were derived above 90% for all the outcomes, which meant that the sample size of 51 patients was sufficient for the statistical analysis.

#### 3.3.1. Pre-Treatment to 3-to-4-Month Patient-Reported Outcomes

The NP NRS and NDI demonstrated statistically significant improvements from pre-treatment to 3-to-4-month re-exams (Figure 6b,c, Table 2). The mean NP NRS score (SD) decreased from 6.0 (±1.0) pre-treatment to 4.7 (±0.8) at 3-to-4 months, resulting in a mean difference of −1.3 (±0.7), which was statistically significant (*p* < 0.001). The NDI (SD) decreased from a pre-treatment mean of 54.3% (±9.3) to 42.6% (±8.5) at 3-to-4 months, resulting in a mean difference of −11.7% (±5.0), which was statistically significant (*p* < 0.001). The mean difference between pre-treatment and 3-to-4-months NDI was −47.5% (±9.0), which was statistically significant (*p* < 0.001). The pre-treatment to 3-to-4-month re-exam NP NRS and NDI did not exceed the MCID thresholds.

#### 3.3.2. Three-to-Four-Month to Post-Treatment Patient-Reported Outcomes

The NP NRS and NDI demonstrated statistically significant improvements from 3-to-4 months to post-treatment (Figure 6b,c, Table 2). The mean NP NRS score (SD) decreased from 4.7 (±0.8) at 3-to-4 months to 1.1 (±0.7) post-treatment, resulting in a mean difference of −3.5 (±0.7), which was statistically significant (*p* < 0.001). The NDI (SD) decreased from a 3-to-4-month mean of 42.6% (±9.3) to 6.8% (±5.5) post-treatment, resulting in a mean difference of −35.8% (±9.0), which was statistically significant (*p* < 0.001). The 3-to-4-month to post-treatment NP NRS and NDI did exceed the MCID thresholds.

#### 3.3.3. Pre-Treatment to Post-Treatment Patient-Reported Outcomes

The NP NRS and NDI demonstrated statistically significant improvements from pre- to post-treatment (Figure 6b,c, Table 2). The mean NP NRS score (SD) decreased from 6.0 (±1.0) pre-treatment to 1.1 (±0.7) post-treatment, resulting in a mean difference of −4.8 (±0.8), which was statistically significant (*p* < 0.001). The NDI (SD) decreased from a pre-treatment mean of 54.3% (±9.3) to 6.8% (±5.5) post-treatment, resulting in a mean difference of −47.5% (±9.0), which was statistically significant (*p* < 0.001). The pre- to post-treatment NP NRS and NDI did exceed the MCID thresholds.

#### 3.3.4. Pearson Correlation Coefficient

**Figure 7:**
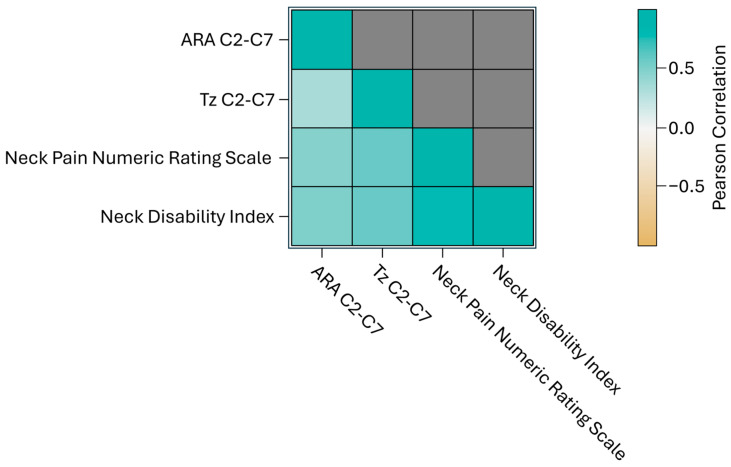
Heat map of Pearson’s correlation coefficient analyses of primary and secondary outcomes. The darker color (green or tan) indicates a higher correlation to positive or negative.

Overall, the results indicated that the CBP^®^ treatment led to significant improvements in ARA C2-C7, Tz C2-C7, NP, and disability due to NP. Pearson’s correlation coefficient (r) was used to assess the analysis, and the results were plotted on a heatmap (Figure 7). The findings revealed strong positive correlations between Tz C2-C7 and NP NRS, Tz C2-C7 and NDI, NP NRS and NDI, ARA C2-C7 and NP NRS, and ARA C2-C7 and NDI (r > 0.7), while a medium correlation was observed between ARA C2-C7 and Tz C2-C7 (0.4 < r < 0.7). Multivariable analyses were deemed inappropriate for this dataset. The improvements in ARA C2-C7 and Tz C2-C7 values, along with improvements in NP NRS and NDI scores, were both statistically and clinically significant, as reflected by *p*-values below 0.001. These findings suggest that the treatment is effective in reducing NP and disability while improving sagittal cervical spinal alignment and posture.

## 4. Discussion

The results of this case series show that Chiropractic BioPhysics^®^ (CBP^®^) is successful in correcting cervical spinal alignment and posture by using MI corrective spinal adjustments, therapeutic spinal exercises, and mechanical spinal traction. This case series shows a positive correlation (direct relationship) between sagittal cervical spinal alignment (cervical lordosis and anterior head translation) and motor vehicle collision (MVC)-induced whiplash-associated disorder (WAD)/cervical acceleration–deceleration (CAD)-related neck pain (NP) and disability metrics. As sagittal cervical spinal alignment improved (by decreasing in value) significant improvements were also observed in MVC-induced WAD/CAD-related NP and disability (also by decreasing in value). Additionally, this case series shows that MVC-induced WAD-/CAD-related NP and disability can still achieve significant improvement or resolution of symptoms after 3-to-4 months of little-to-no change.

In a systematic review of the literature, exercise therapy appears to offer modest benefits for patients with WAD injuries for short-term neck pain and medium-term neck disability, with overall certainty of evidence being low-to-moderate and limited. Exercise therapy spanned from a single primary exercise, cervical rotation, to sets of exercises addressing different functions, including postural adjustments, graded activities, coordination, strength, and aerobic exercises [33]. Across the 27 included studies, positive effects were documented on neck pain and disability, but the direction and size of the effects varied between trials. “The random effects meta-analysis showed a significant effect from exercise on neck pain intensity at 6–8 weeks after treatment, but no significant effect after 10–12 weeks [33].” In contrast, clinical trials with long-term follow-ups that assess CBP^®^ methods and compare against physical therapy protocols show long-term improvement in neck pain and disability with the correction of cervical spinal alignment and posture [34,35,36].

MVC cervical injuries involve damage to capsules, ligaments, intrinsic spinal musculature, and joint anatomies [37]. Studies have reported up to 50% of injured persons involved in a MVC have ongoing NP and disability at long-term follow-up [3]. For the 51 patients in this study, their initial mean NP numeric rating scale (NRS) was 6/10 (moderate pain), and their mean neck disability index (NDI) was 54% (severe disability due to NP) following an MVC trauma. Moderate-to-severe NP and disability are significant and are among the largest contributors to the musculoskeletal global burden of disease (GBD) [1,2].

A correlation between loss of cervical curvature and cervicogenic pain and disability has been reported in patients with MVC-induced WAD/CAD spine injuries [38,39,40,41]. During MVCs, CAD/whiplash alters cervical spine geometry [42]. A retrospective consecutive case series of 41 patients with pre-injury and post-MVC X-ray analysis showed MVCs altered cervical spine alignment with a loss of cervical lordosis (ARA C2-C7) and an increase in cervical sagittal translation (Tz C2-C7) [42]. These findings are consistent with radiographic findings in patients with MVC injuries [38,39,40,41,43]. It is worth noting that the 51 patients in this study were pursuing treatment for cervical spine complaints with an initial mean ARA C2-C7 measuring −10° and a mean Tz C2-C7 measuring 29 mm, which surpass the thresholds for NP and disability (−20° and 25 mm, respectively) [20,21].

It has been theorized that cervical kyphosis or hypolordosis following a MVC is due to muscular spasms in the cervical region [44,45]. However, the only tested evidence that exists shows that hypertonicity and isometric contraction of the cervical spine musculature have no impact on the shape and magnitude of the cervical lordosis [46]. Further, MVC CAD/whiplash occurs faster than cervical muscular reaction time [47,48]. As such, cervical spine muscle spasm or hypertonicity is likely an effect of the WAD/CAD injury and not a cause of loss of cervical lordosis.

Loss of cervical lordosis is a risk factor for, and correlated to, poor long-term health outcomes in patients with MVC-induced WAD/CAD spine injuries [37,38,39,42]. Improvement in cervical lordosis is a desirable clinical goal with significant improvement in short- and long-term health outcomes [34,35,36,49,50,51,52,53]. As such, the failure to assess cervical spinal alignment and posture in the diagnosis of NP and disability can lead to continued unhealthy mechanical stresses and strains on tissues of the spine and subsequent structural, functional, and symptomatic short- and long-term health risks [7,37,38,54].

Trauma physicians need to be aware of cervical spinal alignment and posture parameters prior to considerations for differential diagnosis and patient management. CBP^®^ spinal rehabilitation should be considered if possible and could have a significant impact on the patient outcome and financial burden of the MVC-induced WAD/CAD spine injury. It is noteworthy that the 51 patients in this study achieved a post-treatment mean ARA C2-C7 measuring −22° and a mean Tz C2-C7 measuring 15 mm which meet the thresholds for normal populations (−20° and 15 mm, respectively) [20,21], as well as a post-treatment mean NP NRS of 1/10 (minimal pain) and a mean NDI of 7% (minimal disability due to NP).

### Limitations

This case series provides a clear and well-defined objective and protocol. It includes both objective and subjective clinical outcomes, utilizing valid and reliable quantitative measurements. This case series is retrospective, limiting its applicability to broader patient populations. That said, this series is consecutive, which means that all patients who meet the inclusion criteria are included, while those who met the exclusion criteria were excluded, thereby reducing selection bias. The case series only reports on a low number of patients with a wide age range (18–74 years). That said, post hoc power analysis shows the sample size of 51 patients was sufficient for the statistical analysis. Post hoc power analyses were used, which should be approached with caution, as they were based on observed data rather than predefined assumptions. This study would have benefited from a control group. Future studies would benefit from a prospective design comparing multiple groups (including a control) with stratified age ranges and a larger cohort with a long-term follow-up. The difficulty is being able to work with a population of patients who showed little-to-no change in recovery 3 months following a MVC. Because this is an interventional study involving repeated therapeutic sessions, education, exercise adherence, or increased body awareness may also play a part in patient improvements. This study does not show causation, has no control group, and limits generalizability to apply to other populations. Larger prospective clinical trials with α priori power analysis, control groups, standardization (e.g., consistent number of treatment sessions, treatment duration, etc.), normality testing, and long-term follow-ups are needed to verify these outcomes in patients with cervical DDD and CBP^®^ structural spinal rehabilitation. Future studies should include coronal cervical radiographs, oblique cervical radiographs, flexion and extension cervical radiographs, pre- and post-treatment MRI, and additional measurements (e.g., sagittal and coronal balance, cervical and thoracic morphology, etc.) to further assess biomechanics, alignment, and stability of the cervical spine and their relation to functional and patient-reported outcomes.

## 5. Conclusions

Motor vehicle collision (MVC)-induced whiplash-associated disorder (WAD)/cervical acceleration–deceleration (CAD) spine injuries are prevalent, costly, complicated conditions with neuromusculoskeletal effects that have as much of a chance of leading to ongoing, long-term, chronic neck pain (NP) and disability as does recovery. Thus far, poor recovery has been shown to occur if treatment methods yield little-to-no change in recovery status after 3 months. Chiropractic BioPhysics^®^ (CBP^®^) focuses on restoring healthy alignment and biomechanics to the spine and posture. This case series shows that CBP^®^ spinal rehabilitation may be an effective conservative, non-surgical treatment for MVC-induced WAD/CAD spine injuries, including moderate-to-severe NP and disability. Additionally, based on the results of this study, CBP^®^ may be a solution for MVC-induced WAD/CAD spine injuries with moderate-to-severe NP and disability that have shown little-to-no change by 3 months, which is where other treatments have failed. Through CBP^®^ spinal rehabilitation, improved spinal alignment and postural distortions may negate ongoing, long-term, chronic pain and disability as well as the need for medical or invasive surgical procedures. Future prospective studies involving larger populations, more clinic locations, controlled and experimental groups, and long-term follow-ups, will shed more light on the effectiveness and consistency of CBP^®^ in resolving MVC-induced WAD/CAD spine injuries and the associated functional and symptomatic effects and pathologies.

## Figures and Tables

**Table 1:** Patient demographics and treatment information.

Patient Information	
Patients (n)	51
Males	26 (51.0%)
Females	25 (49.0%)
Mean Height (cm)	171.0 ± 2.6
Mean Weight (kg)	84.7 ± 9.4
Mean Age (y)	42.8 ± 3.6
Mean Treatment Visits (n)	64.5 ± 4.7
Mean Duration of Treatment (wk)	31.8 ± 3.7
Mean Treatment Visits per Week (n)	2.2 ± 0.2

n—number, cm—centimeter, kg—kilogram, y—year, wk—week.

**Table 2:** Comparison of pre-treatment, 3-to-4-month re-exam, and post-treatment patient-reported outcomes.

PROM	Exam	Mean ± SD	Pre vs. 3-to-4		3-to-4 vs. Post		Pre vs. Post		Mean Δ vs. MCID	
			Mean Δ	*p*	Mean Δ	*p*	Mean Δ	*p*	MCID	*p*
NP NRS (n)	Pre	6.0 ± 1.0	−1.3 ± 0.7	**<0.001**	−3.5 ± 0.7	**<0.001**	−4.8 ± 0.9	**<0.001**	2	**<0.001**
	3-to-4	4.7 ± 0.8								
	Post	1.1 ± 0.7								
NDI (%)	Pre	54.3 ± 9.3	−11.7 ± 5.0	**<0.001**	−35.8 ± 9.0	**<0.001**	−47.5 ± 9.0	**<0.001**	15	**<0.001**
	3-to-4	42.6 ± 8.5								
	Post	6.8 ± 5.5								

PROM—patient-reported outcome measure, SD—standard deviation, Pre—pre-treatment values, 3-to-4—3-to-4-month re-exam, Post—post-treatment exam, Δ—difference, *p*—*p*-value, NP NRS—Neck Pain Numeric Rating Scale, n—number, ±—plus or minus, <—less than, NDI—neck disability index, %—percentage. *t*-test was conducted to check the statistical significance. Bolded *p*-value indicated statistical significance within 95% confidence interval (*p* < 0.05).

**Table 3:** Comparison of pre-treatment and post-treatment NLC radiographic measurements.

NLC Radiographic Measurements	Pre CBP^®^ Treatment (95% CI)	Post CBP^®^ Treatment (95% CI)	Mean Difference (95% CI)	*p* Value
ARA C2-C7 (°)	−10.3 ± 2.0	−22.5 ± 2.3	12.2 ± 4.3	**<0.001**
Tz C2-C7 (mm)	28.5 ± 2.0	15.9 ± 1.6	12.6 ± 4.2	**<0.001**

NLC—neutral lateral cervical, CBP^®^—Chiropractic BioPhysics^®^, CI—confidence interval, ARA—absolute rotational angle of measurement, °—degree, Tz—translation in the z-axis, mm—millimeter. *t*-test was conducted to check the statistical significance. Bolded *p*-value indicates statistical significance within 95% confidence interval (*p* < 0.05).

## Data Availability

The data for this study are included in the article. Further inquiries can be directed to the corresponding author.
